# Genetic Engineering *Bacillus thuringiensis* Enable Melanin Biosynthesis for Anti‐Tumor and Anti‐Inflammation

**DOI:** 10.1002/advs.202308506

**Published:** 2024-06-28

**Authors:** Meng Chen, Bingbing Guo, Hui Cheng, Weiyi Wang, Junyi Jin, Yingyi Zhang, Xiaolian Deng, Wenjun Yang, Chenyao Wu, Xiang Gao, Dehong Yu, Wei Feng, Yu Chen

**Affiliations:** ^1^ Materdicine Lab School of Life Sciences Shanghai University Shanghai 200444 P. R. China; ^2^ School of Medicine Shenzhen Campus of Sun Yat‐Sen University Shenzhen 518107 P. R. China; ^3^ Center for Materials Synthetic Biology CAS Key Laboratory of Quantitative Engineering Biology Shenzhen Institute of Synthetic Biology Shenzhen Institute of Advanced Technology Chinese Academy of Sciences Shenzhen 518000 P. R. China; ^4^ School of Environmental and Chemical Engineering Shanghai University Shanghai 200444 P. R. China; ^5^ Oujiang Laboratory (Zhejiang Lab for Regenerative Medicine Vision and Brain Health) Wenzhou Institute of Shanghai University Wenzhou Zhejiang 325088 P. R. China

**Keywords:** anti‐inflammation, engineering bacteria, melanin, photothermal therapy, RONS scavenger

## Abstract

Collaboration between cancer treatment and inflammation management has emerged as an integral facet of comprehensive cancer care. Nevertheless, the development of interventions concurrently targeting both inflammation and cancer has encountered significant challenges stemming from various external factors. Herein, a bioactive agent synthesized by genetically engineering melanin‐producing *Bacillus thuringiensis* (*B. thuringiensis*) bacteria, simultaneously achieves eco‐friendly photothermal agent and efficient reactive oxygen/nitrogen species (RONS) scavenger benefits, perfectly tackling present toughies from inflammation to cancer therapies. The biologically derived melanin exhibits exceptional photothermal‐conversion performance, facilitating potent photonic hyperthermia that effectively eradicates tumor cells and tissues, thereby impeding tumor growth. Additionally, the RONS‐scavenging properties of melanin produced by *B. thuringiensis* bacteria contribute to inflammation reduction, augmenting the efficacy of photothermal tumor repression. This study presents a representative paradigm of genetic engineering in *B. thuringiensis* bacteria to produce functional agents tailored for diverse biomedical applications, encompassing inflammation and cancer therapy.

## Graphical abstract

A genetically engineering melanin‐producing *Bacillus thuringiensis* bacteria‐BMB181, simultaneously achieving eco‐friendly photothermal agent and efficient reactive oxygen/nitrogen species scavenger benefits, perfectly tackling toughies from inflammation to cancer therapies.

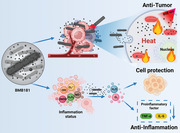

## 1 Introduction

Cancer persists as a substantial global health challenge and ranks among the primary causes of mortality on a global scale.^[^ ^1^ ^]^ Conventional anti‐tumor interventions predominantly concentrate on eradicating tumor cells, often neglecting the interrelated aspect of inflammation, which substantively contributes to the dynamics of tumor growth and progression.^[^ ^2^ ^]^ Indeed, as much as 20% of global cancer‐related fatalities are attributable to underlying infections and inflammation.^[^ ^3^ ^]^ Extensive research and documentation have elucidated the intricate relationship between tumors and inflammation. Chronic inflammation not only fosters tumor initiation, growth, invasion, and metastasis but also compromises the capacity of the immune system to identify and eliminate cancer cells.^[^ ^4^ ^]^ Specifically, inflammatory molecules, encompassing cytokines (tumor necrosis factor‐α and interleukins), chemokines, growth factors, and/or enzymes, establish an inflammatory microenvironment in the vicinity of the tumor, supporting the sustenance and proliferation of the tumor.^[^ ^5^ ^]^ Furthermore, inflammation influences the tumor microenvironment by recruiting immune cells, such as macrophages, which can promote tumor growth.^[^ ^6^ ^]^ The balance between these pro‐inflammatory and anti‐inflammatory responses intricately shapes the trajectory of cancer development. Consequently, targeting both tumor cells and inflammation is indispensable for the efficacy of cancer treatment.

Compared with conventional surgical chemotherapeutic modalities, photothermal therapy (PTT) as a powerful thermal ablation technique destroys cancer cells due to its unique advantages in minimal invasiveness and spatiotemporal selectivity through the accumulation of photothermal agents (PTAs) and precise exposure to localized near‐infrared (NIR) laser irradiation within tumor region.^[^ ^7^ ^]^ Over the past decades, various synthetic PTAs including organic PTAs (such as organic dyes and semiconducting polymers)^[^ ^8^ ^]^ and inorganic PTAs (such as gold‐, carbon‐, and transition metal‐based nanostructures)^[^ ^9^ ^]^ have been extensively developed and utilized in PTT with satisfactory photothermal efficacy. However, severe photobleaching, instability, poor biodegradability, and/or potential toxicity hinder the clinical translation of chemosynthetic PTAs.^[^ ^10^ ^]^ Moreover, the fabrication procedure commonly involves complex physicochemical methods that would be environmentally harmful and have negative effects on the biosafety of PTAs.^[^ ^11^ ^]^

Currently, synthetic biology stands as a rapidly expanding field that amalgamates principles from biology, engineering, and various disciplines to formulate the design and construction of biological modules, systems, and devices.^[^ ^12^ ^]^ A key advantage of synthetic biology in the realm of production lies in its capacity to leverage biological cell factories for the manufacturing of desired products. Unlike chemical processes, biological synthesis typically occurs under mild conditions, resulting in reduced energy consumption and minimized generation of harmful byproducts.^[^ ^13^ ^]^ Notably, the biocompatibility inherent in biological systems enables the production of products that can be directly applied in diverse applications without necessitating intricate purification or modification processes.^[^ ^14^ ^]^ Melanin, as a natural biopolymer widely distributed in various organisms, the analog of which demonstrates remarkable photothermal‐conversion performance.^[^ ^15^ ^]^ Moreover, a substantial phenolic hydroxyl content in melanin can function as a hydrogen donor, effectively counteracting free radicals such as reactive oxygen and nitrogen species, offering it antioxidant performance and playing a crucial role in anti‐inflammation.^[^ ^16^ ^]^ Therefore, melanin and its analog emerge as promising candidates for anti‐tumor and anti‐inflammatory applications. Nevertheless, the natural production of melanin is constrained, and conventional chemical methods suffer from intrinsic limitations.

**Figure 1:**
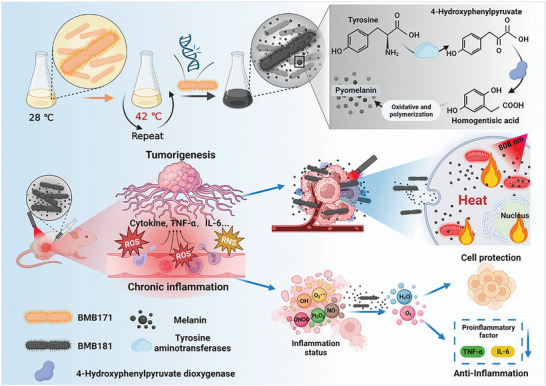
The schematic illustration of BMB181 synthesis and its dual functions on photothermal anti‐cancer and anti‐inflammation. The BMB181‐producing melanin exhibits robust photothermal and RONS scavenging abilities, triggering effective anti‐tumor and anti‐inflammation results.

Herein, melanin with sustained synthesis and release from a genetically modified strain of *Bacillus thuringiensis* (*B. thuringiensis*), designated as BMB181, has been developed as a versatile platform characterized by photothermal and RONS‐scavenging properties, with potential applications in anti‐tumor and anti‐inflammatory treatments (**Figure** **1**). The strong absorption in the near‐infrared (NIR) region, efficient light energy to heat conversion, high photothermal stability, and negligible cytotoxicity of BMB181 are acquired utilizing a mild and environmentally friendly process. An additional significant attribute of BMB181 dispersion is its scavenging capabilities for a broad spectrum of RONS, encompassing hydrogen peroxide (H_2_O_2_), hydroxyl radical (•OH), superoxide anion (O_2_^•−^), nitric oxide (•NO), and peroxynitrite radical (ONOO^−^). Leveraging sustained melanin production, BMB181 exhibits a robust capacity to induce tumor cell death through photonic hyperthermia as well as inhibit inflammatory cytokines such as tumor necrosis factor‐α (TNF‐α) and interleukin 6 (IL‐6) release. This dual functionality positions BMB181 as a distinctive candidate for both anti‐tumor and anti‐inflammatory therapies. This study introduces a living platform utilizing microorganisms for the concurrent treatment of cancer and inflammation, presenting a potentially efficient strategy to counteract tumor progression influenced by inflammatory events.

## 2 Results and Discussion

### 2.1 Preparation and Characterization of BMB181

**Figure 2:**
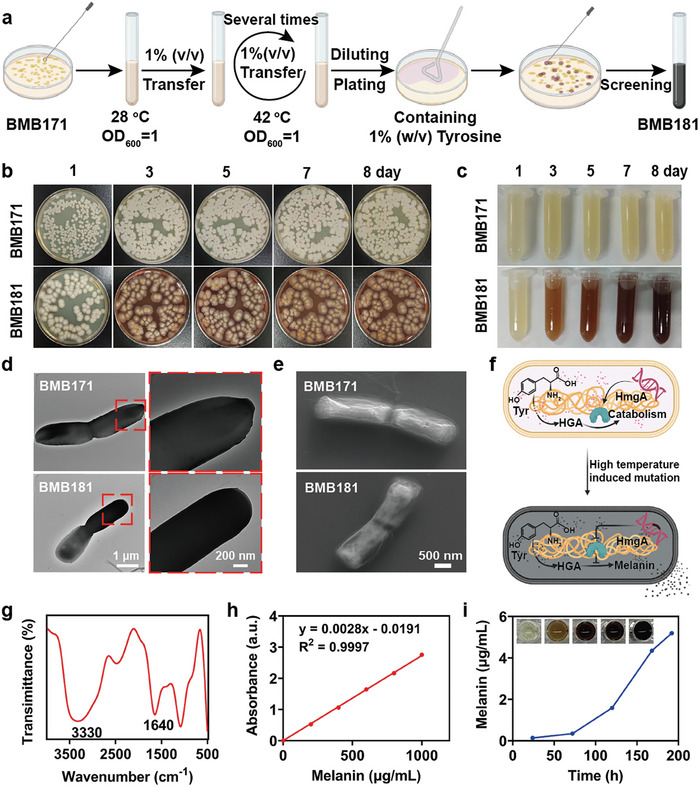
Preparation and characterization of BMB181. a) Schematic illustration of BMB181 strain preparation. Digital photographs of BMB171 and BMB181 in Luria Bertani (LB) agar plates b) and liquid medium c) at the 1st, 3rd, 5th, 7th, and 8th days of cultivation, respectively. d) TEM images of BMB171 and BMB181. e) SEM images of BMB171 and BMB181. f) Schematic diagram of excessive melanin production in BMB181 strain following high‐temperature treatment‐induced HmgA mutation. g) FTIR of melanin produced by BMB181. h) UV–vis absorption standard curve of standard melanin at 400 nm. i) Quantitative analysis of the accumulated melanin concentration by BMB181 over time.

*B. thuringiensis*, a gram‐positive bacterium, was chosen to construct a biosafe living factory for melanin manufacture due to its specific toxicity to pests but almost no harm to humans.^[^ ^17^ ^]^ The BMB181 mutant, characterized by high melanin production, was derived through a series of sequential high‐temperature treatments (42 °C) from the original *B. thuringiensis* strain, BMB171 (**Figure** **2**a). Compared with yellow colonies of BMB171, BMB181 colonies in agar plates appear reddish brown, and the culture medium changes dark over time (Figure 2b,c), indicating that melanin‐producing bacteria have been successfully constructed. Transmission electron microscopy (TEM) (Figure 2d) and scanning electron microscopy (SEM) (Figure 2e; Figure S1, Supporting Information) depict the similar morphology between the BMB171 and BMB181 strains, and there are neglectable influences on the cell viability and bacterial size after bacterial gene mutation (Figure S2, Supporting Information). It is noteworthy that the surface potential of BMB181 (≈−28 mV) is slightly lower than that of BMB171 (≈−24 mV). These subtle differences may be attributed to the synthesis and accumulation of melanin in BMB181 suspension (Figure S3, Supporting Information). High temperature induces mutations in the coding site of the homologous dioxygenase (HmgA) gene, resulting in the spontaneous oxidation and polymerization of the intermediate product, homogentisic acid (HGA), within the tyrosine metabolism pathway (Supplementary Sequence),^[^ ^18^ ^]^ causing the robust melanin generation (Figure 2f). High‐performance liquid chromatography (HPLC) analysis reveals the discernible production and accumulation of HGA in the BMB181 medium compared to that in BMB171 (Figure S4, Supporting Information).

The melanin was further collected through high‐speed centrifugation. TEM images illustrate that the produced melanin manifests as nanoparticles with an average size of ≈100 nm (Figure S5, Supporting Information). Additionally, Fourier transform infrared spectroscopy (FTIR) was employed to investigate the molecular structure of melanin. The peak observed at 3330 cm^−1^ is attributed to the ─OH stretching vibration of melanin, while the strong absorption at 1650 cm^−1^ is assigned to the C═C stretching vibration peak (Figure 2g). Furthermore, the concentration of accumulated melanin reached 5.2 mg mL^−1^ after an 8‐day incubation (Figure 2h,i), thereby confirming the robust capacity of BMB181 for melanin generation.

### 2.2 Photothermal Performance of BMB181

**Figure 3:**
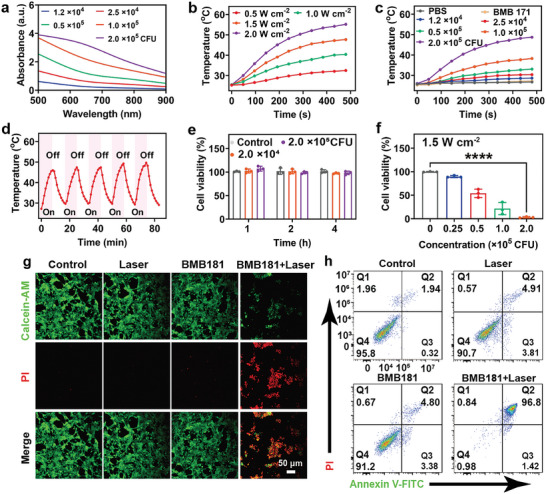
Photothermal performance and photothermal ablation of BMB181. a) UV–vis–NIR absorption spectra of BMB181 dispersions at different concentrations ranging from 1.2 × 10^4^ to 2.0 × 10^5^ CFU. b) Temperature change curves of BMB181 dispersion (2.0 × 10^5^ CFU) upon 808 nm laser irradiation at different power densities (0.5, 1.0, 1.5, and 2.0 W cm^−2^) for 8 min. c) Temperature change curves of BMB171 dispersion (2.0 × 10^5^ CFU) and BMB181 dispersions at different concentrations (0, 1.2 × 10^4^, 2.5 × 10^4^, 0.5 × 10^5^, 1.0 × 10^5^, and 2.0 × 10^5^ CFU) upon 808 nm laser irradiation (1.5 W cm^−2^, 8 min). d) Temperature elevation curves of BMB181 dispersion (2.0 × 10^5^ CFU) of five 808 nm laser on/off cycles (1.5 W cm^−2^). e) Cell viability of HUVEC cells after incubation with BMB181 dispersions at different concentrations (0, 2.0 × 10^4^, and 2.0 × 10^5^ CFU) for 1, 2, and 4 h (*n* = 3). f) Cell viability of 4T1 cells treated with different concentrations of BMB181 (0, 2.5 × 10^4^, 0.5 × 10^5^, 1.0 × 10^5^, and 2.0 × 10^5^ CFU) in the presence of 808 nm laser irradiation (1.5 W cm^−2^, 8 min) (*n* = 3). g) Confocal laser scanning microscopy (CLSM) images of 4T1 cells costaining with Calcein‐AM and PI dyes after different treatments. h) Flow cytometry analysis of apoptotic 4T1 cells after various treatments using the Annexin V‐FITC/PI apoptosis test kit. The data are presented as mean value ± SD. Statistical significance was calculated using Two‐tailed Student's *t*‐test, ^****^ *p* < 0.0001.

The BMB181 dispersion exhibits a broad and intense absorption in the NIR range (**Figure** **3**a), implying the potential for light‐to‐heat conversion. As expected, BMB181 dispersion shows a representative laser power density‐ and concentration‐dependent temperature elevation (Figure 3b,c). The 2.0 × 10^5^ CFU of BMB181 dispersion increases to 48 °C under 808 nm laser irradiation at the laser power density of 1.5 W cm^−2^ for 8 min, while negligible temperature increase of phosphate buffered saline (PBS) and BMB171 dispersion are observed. Thermal images intuitively depict the effective temperature increase of BMB181 exposure to NIR laser irradiation (Figure S6, Supporting Information). Furthermore, almost no significant deterioration of temperature elevation is detected following five successive cycles of laser on‐and‐off irradiation, confirming the photothermal stability of BMB181 (Figure 3d). The photothermal‐conversion efficiency of BMB181 dispersion is quantified at 43.6% (Figure S7, Supporting Information), surpassing the reported polydopamine (PDA) nanoparticles (≈16.6%),^[^ ^19^ ^]^ a melanin analog. Noteworthy is the maintenance of a survival rate exceeding 50% for BMB181 after exposure to heat treatment at 50 °C (Figure S8, Supporting Information), a result consistent with the SYTO9 and propidium iodide (PI) bacterial living and dead staining outcomes (Figure S9, Supporting Information).

Motivated by the remarkable photothermal performance, BMB181 was designed to act as a “living photothermal agent” for cancer cell ablation. Initially, the cytotoxicity of BMB181 was evaluated on both human umbilical vein endothelial cells (HUVEC) and mouse leukemia cell of monocyte macrophage (RAW264.7) using the standard cell counting kit‐8 (CCK‐8) assay. ≈50% of cells survive when bacterial concentration achieves 2.0 × 10^5^ CFU (Figure S10, Supporting Information). Considering the strong reproductive ability of bacteria, the decreased cell viability may be due to the nutrition competition. In short‐time co‐incubation, BMB181 exhibits negligible cytotoxicity when the concentration is below 2.0 × 10^5^ CFU (Figure 3e). Next, 4T1 cells cultured with BMB181 dispersion were exposed to 808 nm laser irradiation. The tumor cell killing efficacy depends on bacterial concentration and laser power density (Figure 3f; Figure S11, Supporting Information), with a noteworthy decrease in cell viability to under 5% achieved using 2.0 × 10^5^ CFU BMB181 with 1.5 W cm^−2^ laser irradiation for 8 min, in contrast to the 100% viability in the control group.

Additionally, the efficacy of photothermal ablation‐induced 4T1 cell death was corroborated through Calcein‐AM and PI co‐staining (Figure 3g; Figure S12, Supporting Information), demonstrating substantial cell ablation efficiency during BMB181‐guided laser treatment, in stark contrast to results observed with either BMB181 or laser treatment alone, where minimal cell death occurs. The apoptosis in 4T1 cells subsequent to photonic hyperthermia treatment was assessed using Annexin V‐FITC/PI staining (Figure 3h). The BMB181 plus laser treatment group displays a significant increase in apoptotic cell ratios, exceeding 95%, providing further evidence of the pronounced photothermal hyperthermia efficacy of BMB181 as the designated living photothermal agent.

### 2.3 RONS‐Scavenging and Anti‐Inflammation

**Figure 4:**
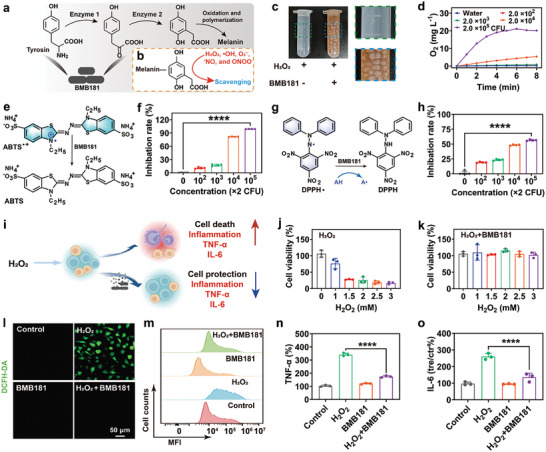
RONS‐scavenging and anti‐inflammation of BMB181. Schematic diagram of melanin synthesis a) and RONS scavenging b). c) Digital photographs of H_2_O_2_ solution treatment with (right) and without (left) BMB181. d) Time‐dependent O_2_ generation by H_2_O_2_ treatment with BMB181 at different concentrations (0, 2.0 × 10^2^, 2.0 × 10^3^, 2.0 × 10^4^, and 2.0 × 10^5^ CFU). e, f) Free radical‐scavenging ability of BMB181 analyzed by ABTS radical cation decolorization assay (*n* = 4). g, h) RNS‐scavenging ability of BMB181 analyzed by DPPH method (*n* = 4). i) Scheme illustration of cytoprotection and anti‐inflammation by BMB181 dispersion. j) Relative viability of HUVEC cells after treatment with H_2_O_2_ at different concentrations (0, 1, 1.5, 2, 2.5, and 3 mm) for 4 h (*n* = 3). k) Relative viability of HUVEC cells after treatment with BMB181 (2.0 × 10^5^ CFU) at different concentrations of H_2_O_2_ (0, 1, 1.5, 2, 2.5, and 3 mm) (*n* = 3). l) CLSM images of HUVEC cells stained with DCFH‐DA after different treatments. m) Flow cytometry analysis of HUVEC cells stained by DCFH‐DA after different treatments. Expression levels of TNF‐α (n) and IL‐6 (o) from the RAW264.7 cells after different treatments (*n* = 3). The data are presented as mean value ± SD. Statistical significance was calculated using Two‐tailed Student's *t*‐test, ^****^ *p* < 0.001.

The specific molecular structures and inter‐molecular interactions enable melanin as an unexceptionable antioxidant for RONS scavenging including hydrogen peroxide (H_2_O_2_), hydroxyl radical (•OH), superoxide anion (O_2_^•−^), nitric oxide (•NO), and peroxynirite (ONOO^−^) (**Figure** **4**a,b).^[^ ^20^ ^]^ BMB181 treated with H_2_O_2_ solution exhibited a significant quantity of bubbles, which suggests that BMB181 can decompose H_2_O_2_ into O_2_ (Figure 4c). Moreover, the production of O_2_ exhibited an upward trend with increasing concentrations of both bacteria and H_2_O_2_ (Figure 4d; Figure S13, Supporting Information). Noteworthy is the observation that, in contrast to a mortality rate exceeding 90% in BMB171, ≈50% of BMB181 survive after 6 h exposure to H_2_O_2_, underscoring its resilience to reactive oxygen species (ROS)‐mediated damage (Figure S14, Supporting Information). BMB181 dispersion demonstrates significant free radical neutralization (Figure 4e–h), with 2.0 × 10^5^ CFU of BMB181 removing ≈96.8% of 2,2′‐azino‐bis(3‐ethylbenzothiazoline‐6‐sulfonic acid) diammonium salt radical cation (ABTS^•+^) and 56.5% of 2,2‐di(4‐tert‐octylphenyl)−1‐picrylhydrazyl (DPPH) radicals. Moreover, electron spin‐resonance spectroscopy (ESR) spectra show that •OH is removed by BMB181 effectively (Figure S15, Supporting Information), with a scavenging rate exceeding 90% at a bacterial concentration of 2.0 × 10^5^ CFU (Figure S16, Supporting Information). Apart from that, O_2_^•−^, a potent oxidizing agent, can be cleared by BMB181 as well (Figure S17, Supporting Information). The predominant mechanism underlying the elimination of RONS by BMB181 can be primarily ascribed to the presence of phenolic hydroxyl structures in the intermediate molecules formed during melanin synthesis (Figure 4b), which act as hydrogen donors to combine with free radicals, offering the bacteria with a potent antioxidant capacity.

Oxidative stress can cause cell injury and chronic inflammation, and even long‐term exposure to high levels of pro‐oxidant factors can lead to the development of cancer.^[^ ^21^ ^]^ Motivated by the compelling antioxidative properties, we proceeded to investigate the cytoprotective and anti‐inflammatory effects of BMB181 (Figure 4i). As a small non‐charged molecule that can easily pass through the cytomembrane and localize in organelles, H_2_O_2_ results in concentration‐dependent cytotoxicity in which cell viability declines from ≈76.2% to ≈16.9% when H_2_O_2_ concentration rises from 1 to 3 mm (Figure 4j). However, BMB181 can effectively reverse H_2_O_2_‐induced oxidative stress to preserve cell survival (Figure 4k). To delve further into the cytoprotective mechanism of BMB181, 2′,7′‐dichlorodihydrofluorescein diacetate (DCFH‐DA) was employed to assess intracellular ROS levels (Figure 4l). Compared to the control and BMB181 treatment groups, stronger intracellular fluorescence is clearly observed in cells following H_2_O_2_ treatment, while BMB181 can effectively diminish H_2_O_2_‐triggered intense fluorescent signals, demonstrating that BMB181 protects cells from oxidative stress‐induced cytotoxicity, which is further verified by flow cytometry analysis (Figure 4m).

As the main participants in the occurrence and development of inflammation, RAW264.7 macrophages were then used to evaluate the anti‐inflammation of BMB181. Untreated cells exhibit a spherical morphology with smooth cell edges and an absence of pseudopodia (Figure S18, Supporting Information). However, H_2_O_2_ stimulation results in the formation of pseudopodia in RAW264.7 cells, suggesting that oxidative stress causes the activation of macrophages. Notably, BMB181 can successfully impede the pseudopodia formation in RAW264.7 cells. Furthermore, the levels of two crucial pro‐inflammatory cytokines, TNF‐α and IL‐6, decrease significantly in the H_2_O_2_ + BMB181 group compared with that of the H_2_O_2_‐treated group, approaching levels akin to those observed in the control (Figure 4n,o). Taken together, BMB181 can inhibit cell damage from oxidative stress, prevent immune cell activation, and consequently down‐regulate the release of pro‐inflammatory cytokines, featuring high anti‐inflammation efficiency.

### 2.4 Anti‐Tumor and Anti‐Inflammatory Effects In Vivo

In view of the biosafety considerations pertinent to bacterial therapy, the in vivo biosafety of BMB181 was evaluated. The results indicate a significant clearance of bacteria in tumor tissues within 5 days following BMB181 injection, culminating in complete elimination by the 7th day (Figure S19, Supporting Information). Importantly, there is no observable accumulation of BMB181 in the major organs or blood, which highlights the favorable biosafety of BMB181 in vivo. Furthermore, hematoxylin‐eosin (H&E) staining of main organs, including the heart, liver, spleen, lung, and kidney, demonstrates no detectable tissue damage after BMB181 injection (Figure S20, Supporting Information). Additionally, comprehensive blood biochemistry and blood routine analyses were conducted on blood samples post‐BMB181 injection to assess peripheral blood, liver, and kidney functions (Figure S21, Supporting Information). The results unambiguously suggest that BMB181 administration exerts no significant alterations in hematological and biochemical parameters, confirming the lack of acute inflammatory responses and major toxicity induced by BMB181.

**Figure 5:**
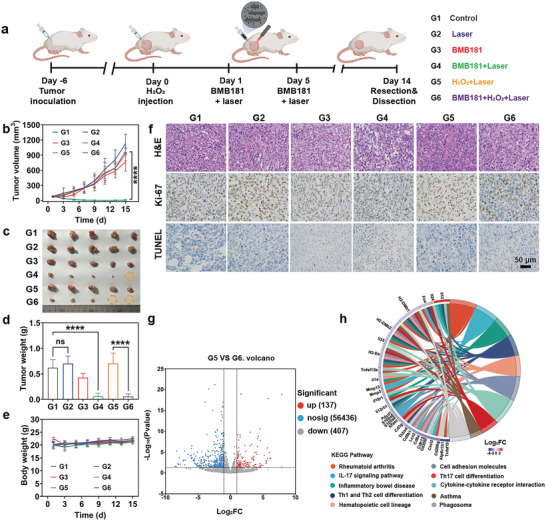
BMB181‐mediated photothermal ablation and anti‐inflammation in vivo. a) Schematic illustration of BMB181‐mediated cancer therapy on 4T1 tumor‐bearing mice. b) Average tumor growth curves of 4T1 tumor‐bearing mice after different treatments (*n* = 5). c) Digital photographs of the dissected tumors from mice after different treatments (*n* = 5). d) Tumor weights of mice after different treatments (*n* = 5). e) Bodyweight changes of mice after different treatments (*n* = 5). f) H&E, Ki‐67, and TUNEL stained pathological changes in tumor tissues after different treatments. g) Volcano map showing the identified upregulated and downregulated genes between H_2_O_2_ + Laser group (G5) and BMB181 + H_2_O_2_ + Laser group (G6). h) KEGG pathways enrichment analysis of the identified differentially expressed genes. The data are presented as mean value ± SD. Statistical significance was calculated using one‐way analysis of variance (ANOVA) with Tukey multiple comparison, ^****^ *p* < 0.0001.

Next, the anti‐tumor efficacy of BMB181 was systematically investigated in vivo (**Figure** **5**a), which were randomly divided into six groups: i), Control group; ii), Laser group; iii), BMB181 group; iv), BMB181 + Laser group, v), H_2_O_2_ + Laser group, and vi), BMB181 + H_2_O_2_ + Laser group. Following the administration of bacteria, tumors were exposed to 808 nm laser irradiation, and the body weights and tumor volumes were measured and recorded every other day during 14 days of treatment. Compared with the only laser treatment group, the temperature of the tumor region could be increased to 50 °C after laser irradiation for 5 min post‐injection of BMB181 (Figure S22, Supporting Information). Similarly, the temperature in the BMB181 + H_2_O_2_ + Laser group rapidly rises under 808 nm laser illumination. It is noteworthy that the BMB171 + Laser treatment fails to induce significant photothermal effects, underscoring that the remarkable photothermal efficacy of BMB181 is primarily attributed to the efficient release of melanin. The tumor growth in mice treated with BMB181 + Laser and BMB181 + H_2_O_2_ + Laser is substantially suppressed (Figure 5b–d; Figures S23 and S24, Supporting Information), with tumor inhibition rates reaching 90% (Figure S25, Supporting Information), resulting from the robust photothermal conversion capacity, exhibiting satisfactory tumor suppression effects. In addition, no significant weight loss was observed during the treatment period (Figure 5e).

Furthermore, H&E staining, Ki‐67 staining, TdT‐mediated dUTP‐biotin nick end labeling (TUNEL) staining, and caspase‐3 staining were performed to evaluate the antitumor effects of BMB181‐mediated photothermal hyperthermia (Figure 5f; Figure S26, Supporting Information). Compared with control and laser groups, high‐temperature induced tumor tissue destruction and cell nucleus shrinkage are observed in the BMB181 + Laser group and BMB181 + H_2_O_2_ + Laser group. Ki‐67 staining manifests that BMB181‐mediated photothermal treatments inhibit tumor cell proliferation. TUNEL and caspase‐3 staining demonstrate that BMB181‐mediated photothermal ablation dramatically results in tumor cell apoptosis. The anti‐inflammatory efficacy of BMB181 was evaluated. Notably, the injection of H_2_O_2_ results in a significant increase in immune cell infiltration (Figure 5f), indicating intense inflammation. However, subsequent to PTT, the melanin produced by BMB181 markedly reduced immune cell infiltration and facilitated the regeneration of collagen fibers (Figure S27, Supporting Information). Furthermore, the levels of TNF‐α and IL‐6 in tumor tissues are significantly elevated in the group treated with H_2_O_2_, whereas the administration of BMB181 leads to a substantial decrease in these pro‐inflammatory factors (Figures S28 and S29, Supporting Information), which underscores the ability of BMB181 to mitigate the overproduction of proinflammatory cytokines, highlighting its potential as an effective agent in reducing inflammation within the tumor microenvironment.

To further identify the potential mechanism underlying BMB181‐mediated anti‐tumor and anti‐inflammatory therapies in 4T1 tumor‐bearing mice, RNA sequencing (RNA‐seq) analysis was conducted. Among 56455 mRNA transcripts between G1 and G5, 525 significant differentially expressed genes (DEGs) are identified, including 325 upregulated genes and 200 downregulated genes (| Fold Change | ≥ 2, *p* < 0.05) (Figure S30, Supporting Information). For those DEGs, the genes relate to pro‐inflammation response, including interleukin‐1β (IL1b), C‐X‐C motif chemokine 5 (Cxcl5), and interleukin 17 receptor B (IL17rb), are significantly up‐regulated. In addition, the genes of Fos and chemokine (C‐C motif) ligand 5 (Ccl5) related to tumor cell growth, invasion, and metastasis are up‐regulated, demonstrating that inflammation could promote tumor progress (Figure S31, Supporting Information). Subsequently, tumor tissues of the G5 and G6 groups were further analyzed. Among the identified 544 significant DEGs, there are 137 upregulated genes and 407 downregulated genes (| Fold Change | ≥ 2, *p* < 0.05) (Figure 5g), implying that the BMB181‐based therapy remodels the transcriptome of tumor tissues.

Kyoto Encyclopedia of Genes and Genomes (KEGG) analysis in metabolic pathways reflects main activation pathways including the interleukin‐17 (IL‐17) signaling pathway, T helper type 17 (Th17) cell differentiation, and necroptosis (Figure S32, Supporting Information). In these pathways, most of the down‐regulation genes are related to pro‐inflammatory reactions, suggesting the relieved inflammation induced by H_2_O_2_ under the BMB181 administration. Moreover, the down‐regulation genes such as ATPase H^+^ transporting V1 subunit B1 (Atp6v1b1) and phospholipase A2 group IV C (Pla2g4c) associated with oxidative phosphorylation and cell metabolism and the up‐regulated genes such as WNT inhibitory factor 1 (Wif1) associated with tumor suppression reveal that BMB181 + Laser treatment is effective against cancer (Figure S33, Supporting Information). To elaborate on the biological functions, the enriched chord diagram of the KEGG pathway for the selected pathways is correlated with immune regulation and cell migration to reveal the therapeutic mechanisms after BMB181‐mediated photothermal hyperthermia and anti‐inflammation therapies (Figure 5h). These findings further substantiate the efficacy of the dual treatment approach based on BMB181 in inhibiting tumor development and reducing inflammation.

## 3 Conclusion

In summary, we have developed a melanin‐producing bacterium BMB181 with satisfactory photothermal therapeutic potency and excellent RONS‐scavenging capacity, achieving anti‐tumor and anti‐inflammation therapies. The BMB181 is derived from a sequential high‐temperature induction gene mutant from the BMB171 strain. The potent photothermal‐conversion ability enables BMB181 dispersion to successfully induce thermo‐killing of tumor cells. Furthermore, based on the phenolic hydroxy of melanin, BMB181 effectively attenuates ROS‐induced cellular damage and reduces the expression level of pro‐inflammatory factors in vitro. The H_2_O_2_‐induced 4T1 subcutaneous tumor mice model with local inflammation was chosen due to its ease of establishment and study of tumor ablation and inflammation mitigation. A peritumoral injection of BMB181 followed by laser treatment significantly suppresses the tumor progression. More importantly, tumor‐localized inflammation is concurrently reduced, as evidenced by decreased levels of pro‐inflammatory factors, including TNF‐α and IL‐6, in tumor tissues at the end of the therapeutic period. Collectively, the rationally engineered BMB181 can serve as a distinct living therapeutic platform to overcome both inflammation and cancer progression, paving the way to reduce the promoting effect of inflammation on cancer progression/recurrence and correspondingly enhance the efficacy of anti‐tumor therapies.

## 4 Experimental Section

### Strains, Cells, and Materials

BMB171 and BMB181 strains were obtained from the Institute of Synthetic Biology, Shenzhen Institute of Advanced Technology, Chinese Academy of Sciences. Human umbilical vein endothelial cell (HUVEC) lines, 4T1 murine breast cancer cell lines, and mouse monocyte macrophage leukemia cell lines (RAW264.7) were purchased from the cell bank of the Chinese Academy of Sciences (CBCAS, Shanghai). Standard melanin was purchased from Aladdin (Shanghai Naicheng, China). The total antioxidant capacity test kit and reactive oxygen species assay kit were both from Beyotime (Shanghai, China). 1,1‐diphenyl‐2‐picrylhydrazyl (DPPH) was purchased from Shanghai Yuanye Bio‐Technology Co., Ltd. Hydroxyl radical scavenging ability test kit was obtained from Solarbio, China. SYTO9 and propidium iodide (PI) live/dead bacterial double stain kit was from Invitrogen. Roswell park memorial institute‐1640 (RPMI1640) and Dulbecco's modified Eagle's medium (DMEM) were both obtained from Sangon (Shanghai, China). Fetal bovine serum (FBS) was purchased from Gibco (Shanghai, China). Cell counting kit‐8 (CCK‐8) was obtained from Dojindo China Co., Ltd. The Mouse TNFA/TNF alpha enzyme‐linked immunosorbent assay (ELISA) kit was obtained from Boster Biological Technology Co., Ltd. (Wuhan, China). Mouse interleukin 6 (IL‐6) ELISA kit was purchased from ABclonal (Wuhan, China). Ultrapure water (Unique‐R10, 18.2 MΩ) was implemented in the experiments.

### Apparatus and Characterization

The molecular structure of BMB181‐producing melanin was analyzed by VERTEX70 Fourier Transform Infrared (FTIR) spectrum instrument. Zetasizer Nano ZS90 Malvern particle characterization system was used to measure the hydrodynamic diameters and potentials of BMB171 strain, BMB181 strain, and melanin. The microstructure of BMB171 and BMB181 was observed by G300 scanning electron microscope and JEM‐2100F transmission electron microscope. The microstructure of melanin released by BMB181 was inspected via JEM‐2100F transmission electron microscope. The Axio Imager 2 fluorescence microscopy was used to monitor fluorescence images of bacterial living dead staining. Ultraviolet–visible (UV–vis) absorbance spectra were detected through UV‐1800 UV–vis scanning spectrometer. Free radical reaction tests were detected through JEOL‐FA200 electron spin resonance (ESR) spectrometer. Confocal laser scanning microscopy (CLSM) images were observed by an Olympus FV1200 confocal laser scanning microscope.

### Bacterial Culture and Growth

BMB171 and BMB181 were cultured in Luria Bertani (LB) medium (10 g L^−1^ tryptone, 10 g L^−1^ NaCl, and 5 g L^−1^ yeast extract, 1% (w/v) tyrosine) and placed in a culture shaker at a constant speed of 200 rpm for 28 °C aerobic growth. To detect bacterial growth, BMB171 and BMB181 were diluted to an initial optical density at 600 nm (OD_600_) of ≈0.2 and incubated in LB medium for 28 °C aerobic growth with a constant speed of 200 rpm. OD600 value was recorded every 2 h by UV.

### Melanin Generation and Quantitation

BMB171 and BMB181 with an initial optical density at 600 nm of ≈0.2 were incubated in LB medium for 28 °C aerobic growth with a constant speed of 200 rpm. The color changes of the bacterial dispersion were recorded on different days. To quantify the melanin, BMB181 dispersion was centrifugated, and the OD_400_ of the collected supernatant was measured at different time points by UV.

### Cell Culture

The 4T1 cells were cultured in RPMI‐1640 medium containing 10% (v/v) of FBS. HUVEC cells and RAW264.7 cells were cultured in DMEM supplemented with 10% (v/v) of FBS. Cells were cultured in the cell culture flask (25 cm^2^) and placed in a cell incubator with 5% CO_2_ at 37 °C.

### Cell Toxicity

HUVEC and 4T1 cells were seeded in 48‐well plates for 24 h culture. Then, different concentrations of BMB181 dispersions (0, 2.0 × 10^4^, and 2.0 × 10^5^ CFU) were incubated with cells for 1, 2, and 4 h, respectively. At last, the cell viability was measured by a standard CCK‐8 assay. The absorbance at 450 nm of each well was recorded by a microplate reader, and the cell viability was calculated by following Equation (1).

(1) $$\mathrm{Cell}\;\mathrm{viab}\mathrm{ility}\left(\%\right)=\left(OD_{\mathrm{treat}}-OD_{\mathrm{blank}}\right)÷\left(OD_{\mathrm{cont}\mathrm{rol}}-OD_{\mathrm{blank}}\right)\times 100\%$$

where *OD*_treat_ refers to the absorbance of different treatment wells, *OD*_blank_ is the absorbance of blank wells, and *OD*_control_ means the absorbance of wells without any treatment.

### Cell Living/Dead Staining

4T1 cells were seeded into confocal dishes for 24 h culture. Then, cells were treated with different processes, including Control, Laser, BMB181, BMB181 + Laser. For PTT, the groups in laser and BMB181 (2.0 × 10^5^ CFU) + Laser were irradiated by 808 nm laser with the power density at 1.5 W cm^−2^ for 8 min. After 4 h incubation, cells were costained with calcein acetoxymethyl ester (Calcein AM, 5 µM) and propidium iodide (PI, 10 µM) for 30 min according to the instructions. The ultimate images were observed by CLSM.

### Cell Apoptosis Analysis

The 4T1 cells were seeded in 6‐well plates for 24 h culture. Then, cells were treated with laser (808 nm, 1.5 W cm^−2^), BMB181 (2.0 × 10^5^ CFU), and BMB181 plus laser (2.0 × 10^5^ CFU, 808 nm, 1.5 W cm^−2^), respectively. The untreated cells were as control. After different treatments, cells were cultured for another 4 h. Then, cells were trypsinized, washed, and centrifuged at 1000 rpm for 5 min. Subsequently, cells were collected and stained with Annexin V‐FITC (10 µM) and PI (10 µM) for 15 min at room temperature in the dark. Lastly, the results were analyzed by flow cytometry immediately.

### RONS Scavenging

H_2_O_2_ scavenging of BMB181 dispersion was analyzed by measuring oxygen generation using a dissolved oxygen meter (JPSJ‐605F) under continuous stirring. The oxygen detection was conducted in different concentrations of BMB181 dispersions (0, 2.0 × 10^2^, 2.0 × 10^3^, 2.0  × 10^4^, and 2.0 × 10^5^ CFU), and different concentrations of H_2_O_2_ (0, 0.1, 0.5, 1.0, and 5.0 mm).

The free radical scavenging activity was evaluated by ABTS (2, 2′‐azinobis (3‐ethylbenzothiazoline‐6‐sul‐fonate)) radical cation decolorization assay according to the instructions provided by manufacturers. The general reactive nitrogen species scavenging activity was measured by 2, 2‐di‐(4‐tert‐octylphenyl)−1‐picrylhydrazyl (DPPH) method. To be specific, different concentrations of BMB181 dispersions (0, 2.0 × 10^2^, 2.0 × 10^3^, 2.0 × 10^4^, and 2.0 × 10^5^ CFU) were mixed with DPPH and incubated in the dark at room temperature for 30 min. Subsequently, the mixtures were centrifuged at 5000 rpm for 10 min. The supernatants were extracted for the detection of the absorbance value at 517 nm.

The hydroxyl radical scavenging ability was detected through ESR experiments and hydroxyl radical scavenging capacity assay. To be specific, hydroxyl radical was derived from the Fenton reaction. The reaction solution containing BMB181 or not was incubated with a 5,5‐dimethyl‐1‐pyrroline N‐oxide (DMPO) spin–trapping agent for hydroxyl radical capture, respectively. The ESR spectra were recorded. The hydroxyl radical scavenging capacity assay was carried out according to the procedure provided by manufacturers.

Superoxide radical scavenging was evaluated through ESR experiments. Superoxide radical was obtained from the xanthine and xanthine oxidase reaction system. The reaction solution containing BMB181 or not was incubated with a DMPO spin–trapping agent for superoxide radicals capture, respectively. The ESR spectra were recorded.

### Cytoprotection and Anti‐Inflammation In Vitro

HUVEC cells were seeded into 48‐well plates for 24 h culture. Different concentrations of H_2_O_2_ (0, 1.0, 1.5, 2.0, 2.5, and 3.0 mm) were coincubated with HUVEC cells under BMB181 dispersion (2.0 × 10^5^ CFU) supplementation or not. After 4 h coincubation, the cell viability was evaluated by the CCK‐8 assay. The final absorbance value at 450 nm of each well was recorded by a microplate reader. The cell viability was calculated by Equation (1).

ROS scavenging of BMB181 dispersion was measured by a DCFH‐DA probe. HUVEC cells were incubated with 1 mL DCFH‐DA solution diluted by DMEM medium without FBS at the ratio of 1:1000. After 20 min incubation, cells were treated differently, including H_2_O_2_ (3 mm), BMB181 dispersion (2.0 × 10^5^ CFU), and H_2_O_2_ (3 mm) together with BMB181 dispersion (2.0 × 10^5^ CFU). The untreated cells were as a control group. After 1 h incubation, the fluorescence intensity was recorded by CLSM as quickly as possible.

Evaluation of BMB181 inhibition of RAW264.7 macrophage polarization. RAW264.7 macrophages were seeded into 48‐well plates for 24 h culture. Then cells were treated differently, including control, H_2_O_2_ (1 mm), BMB181 (2.0 × 10^5^ CFU), and H_2_O_2_ + BMB181 (1 mm, 2.0 × 10^5^ CFU). At last, the shape changes of RAW264.7 cells after different treatments were observed with a microscope.

The tumor necrosis factor‐α (TNF‐α) and IL‐6 levels were evaluated by commercial enzyme‐linked immunosorbent assay kits. HUVEC and RAW264.7 cells were pre‐cultured in 96‐well plates for 24 h before use. Then, HUVEC cells were treated differently as follows: H_2_O_2_ (3 mm), BMB181 (2.0 × 10^5^ CFU), and H_2_O_2_ + BMB181 (3 mm, 2.0 × 10^5^ CFU) for 2 h. The untreated cells were as a control group. Next, transferred the supernatants of HUVEC cells after different treatments to a 96‐well plate containing RAW264.7 cells, and culture for another 12 h. Finally, the supernatants of RAW264.7 cells were collected and TNF‐α and IL‐6 levels were measured by using commercial ELISA assay kits according to the protocol provided by manufacturers.

### Animal Model

All experimental animals were conducted in strict accordance with the guidelines of the Institutional Animal Care and Use Committee of Shanghai University (Approval number: ECSHU 2022–053). To construct tumor‐bearing mice model, female BALB/c mice (6‐8 weeks old) were subcutaneously injected with 150 µL 4T1 cells (1 × 10^6^ cells) into the right flank. For inducing inflammation response in tumor tissue, 100 µL H_2_O_2_ (1 mm) was injected into the tumor one day before the start of treatment. All mice were raised in specific pathogen‐free (SPF) class environment facility (temperature: 20 ± 3 °C, relative humidity: 55 ± 15%).

### Photothermal Effects of BMB181 In Vivo

BMB181 of 100 µL (2.0 × 10^5^ CFU) was peritumoral injected into 4T1 tumor‐bearing mice. Then, an 808 nm laser with a power density of 1.5 W cm^−2^ was irradiated areas where BMB181 injection. Temperature changes were recorded by the Fotric 225S‐L24 infrared thermal imager. Photothermal images at different times were recorded and temperature‐rising curves were drawn by the software of GraphPad Prism 9.0.

### Anti‐Tumor Treatment In Vivo

When the volume of tumors reached 75 mm^3^, 4T1 tumor‐bearing mice were divided into six groups randomly (*n* = 5) for diverse treatments: i) Control; ii) Laser (808 nm, 1.5 W cm^−2^, 5 min); iii) BMB181 (2.0 × 10^5^ CFU); iv) BMB181 + Laser (2.0 × 10^5^ CFU, 808 nm, 1.5 W cm^−2^, 5 min); v) H_2_O_2_ + Laser (1 mm, 808 nm, 1.5 W cm^−2^, 5 min); and vi) BMB181 + H_2_O_2_ + Laser (2.0 × 10^5^ CFU, 1 mm, 808 nm, 1.5 W cm^−2^, 5 min). The treatment manipulation was carried out on day 1 and day 5, respectively. During the therapeutic period, mice's tumor volume and body weight were measured every other day. Tumor volume was calculated by following Equation (2):

(2) $$Tumor\;volume\left(mm^{3}\right)=tumor\;length\;\times \;tumor\;width^{2}÷2$$

At the end of treatment, the mice were euthanized by cervical dislocation method. At the same time, the tumor tissues of mice were collected for histological and immunofluorescence analysis.

### Statistical Analysis

The data calculated in the experiment were all presented as mean ± SD, and each data quantitative evaluation was repeated at least three times. Two‐tailed Student's *t*‐test was performed to evaluate the statistical significance between the groups. One‐way analysis of variance (ANOVA) with Tukey multiple comparison test was conducted to evaluate the statistical significance among more than two groups. The results were significant with signs of ^**^p < 0.01, ^***^p < 0.001, and ^****^p < 0.0001. All data were analyzed by Origin 2021 and Graphpad Prism 9.0.

## Conflict of Interest

The authors declare no conflict of interest.

## Supporting information

Supporting Information （The Supplementary Information has been modified and uploaded in PDF and Word text formats.）

## Data Availability

The data that support the findings of this study are available from the corresponding author upon reasonable request.
