# Supplementary material for: Genetic Engineering Bacillus thuringiensis Enable Melanin Biosynthesis for Anti‐Tumor and Anti‐Inflammation
Source: Adv Sci (Weinh). 2024 Jun 28;11(36):2308506. doi: 10.1002/advs.202308506 (PMC11423088; doi:10.1002/advs.202308506)
Supplement: Supplementary file 1 — Supporting Information （The Supplementary Information has been modified and uploaded in PDF and Word text formats.） [file ADVS-11-2308506-s001.pdf]

ADVANCED SCIENCE
Open Access

Supporting Information

for *Adv. Sci.*, DOI 10.1002/advs.202308506

Genetic Engineering *Bacillus thuringiensis* Enable Melanin Biosynthesis for Anti-Tumor and Anti-Inflammation

Meng Chen, Bingbing Guo, Hui Cheng, Weiyi Wang, Junyi Jin, Yingyi Zhang, Xiaolian Deng, Wenjun Yang, Chenyao Wu, Xiang Gao, Dehong Yu*, Wei Feng* and Yu Chen**

Supporting Information

for *Adv. Sci.*, DOI 10.1002/advs.202308506

Genetic Engineering *Bacillus thuringiensis* Enable Melanin Biosynthesis for Anti-tumor and Anti-inflammation

Meng Chen, Bingbing Guo, Hui Cheng, Weiyi Wang, Junyi Jin, Yingyi Zhang, Xiaolian Deng, Wenjun Yang, Chenyao Wu, Xiang Gao*, Dehong Yu*, Wei Feng* and Yu Chen*

Supporting Information

Genetic Engineering *Bacillus thuringiensis* Enable Melanin Biosynthesis for Anti-tumor and Anti-inflammation

Meng Chen[1], Bingbing Guo[1], Hui Cheng[1], Weiyi Wang[1], Junyi Jin[1], Yingyi Zhang[2,3], Xiaolian Deng[2,3], Wenjun Yang[3], Chenyao Wu[1], Xiang Gao[3]*, Dehong Yu[1]*, Wei Feng[1,4,5,]*, Yu Chen[1,4,5,]*

Affiliations:

[1]Materdicine Lab, School of Life Sciences, Shanghai University, Shanghai, 200444, P. R. China

[2]School of Medicine, Shenzhen Campus of Sun Yat-Sen University, Shenzhen, 518107, P. R. China

[3]Center for Materials Synthetic Biology, CAS Key Laboratory of Quantitative Engineering Biology, Shenzhen Institute of Synthetic Biology, Shenzhen Institute of Advanced Technology, Chinese Academy of Sciences, Shenzhen, 518000, P. R. China

[4]School of Environmental and Chemical Engineering, Shanghai University, Shanghai, 200444, P. R. China

[5]Oujiang Laboratory (Zhejiang Lab for Regenerative Medicine, Vision and Brain Health) Wenzhou Institute of Shanghai University, Wenzhou, Zhejiang, 325088, P. R. China

* These are corresponding authors.

E-mail:
Xiang Gao, gaoxiang@siat.ac.cn
Dehong Yu, dehongyu@shu.edu.cn
Wei Feng, fengw@shu.edu.cn
Yu Chen, chenyuedu@shu.edu.cn

Keywords: anti-inflammation; engineering bacteria; melanin; photothermal therapy; RONS scavenger

SUPPORTING INFORMATION

Table of Contents

Results and Discussion **3**
Figure S1. 3
Figure S2. 3
Figure S3. 4
Figure S4. 4
Figure S5. 5
Figure S6. 5
Figure S7. 6
Figure S8. 6
Figure S9. 7
Figure S10. 7
Figure S11. 8
Figure S12. 8
Figure S13. 9
Figure S14. 9
Figure S15. 10
Figure S16. 10
Figure S17. 11
Figure S18. 11
Figure S19. 12
Figure S20. 12
Figure S21. 13
Figure S22. 13
Figure S23. 14
Figure S24. 14
Figure S25. 15
Figure S26. 15
Figure S27. 16
Figure S28. 16
Figure S29. 17
Figure S30. 17
Figure S31. 18
Figure S32. 18
Figure S33. 19
Supplementary Sequence 20
Reference **22**
Author Contributions **22**

Results and Discussion

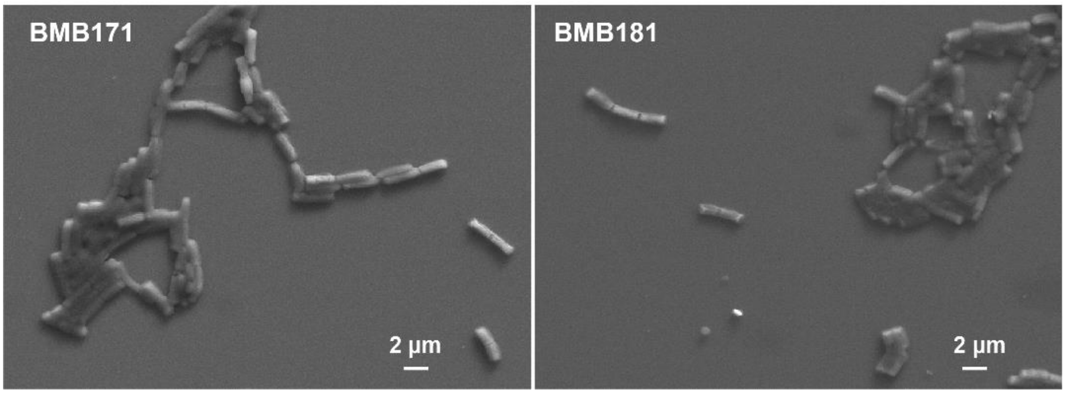

Figure S1. SEM images of BMB171 and BMB181.

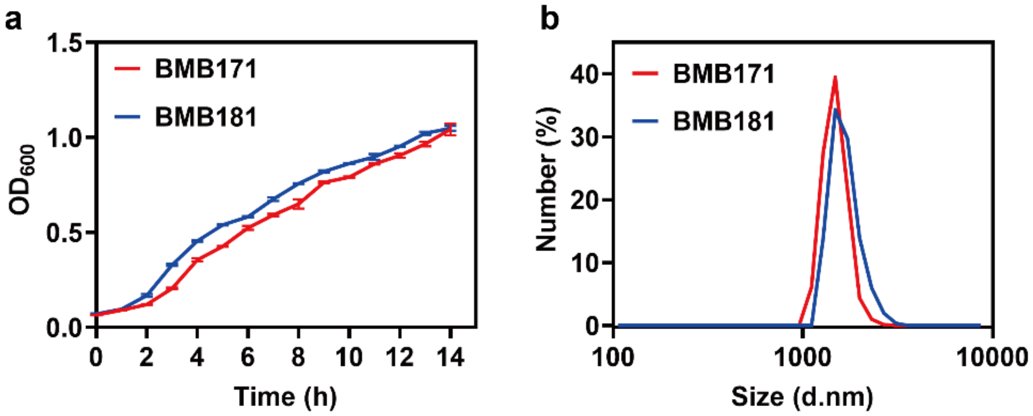

Figure S2. (a) The growth curves of BMB171 and BMB181 during 14 h culturing (n = 3). (b) The DLS data of BMB171 and BMB181. The data are presented as mean value ± SD.

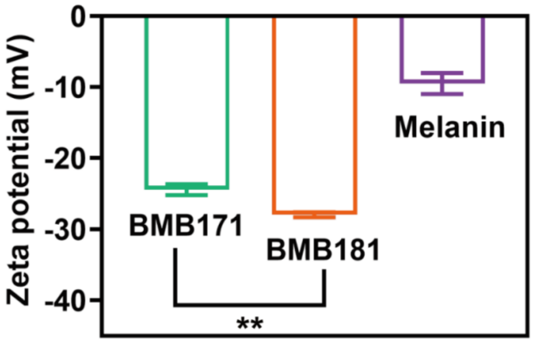

Figure S3. Zeta potentials of BMB171, BMB181, and produced melanin (n = 3). The data are presented as mean value ± SD. Statistical significance was calculated using Two-tailed Student's t-test, $^{**}p < 0.01$.

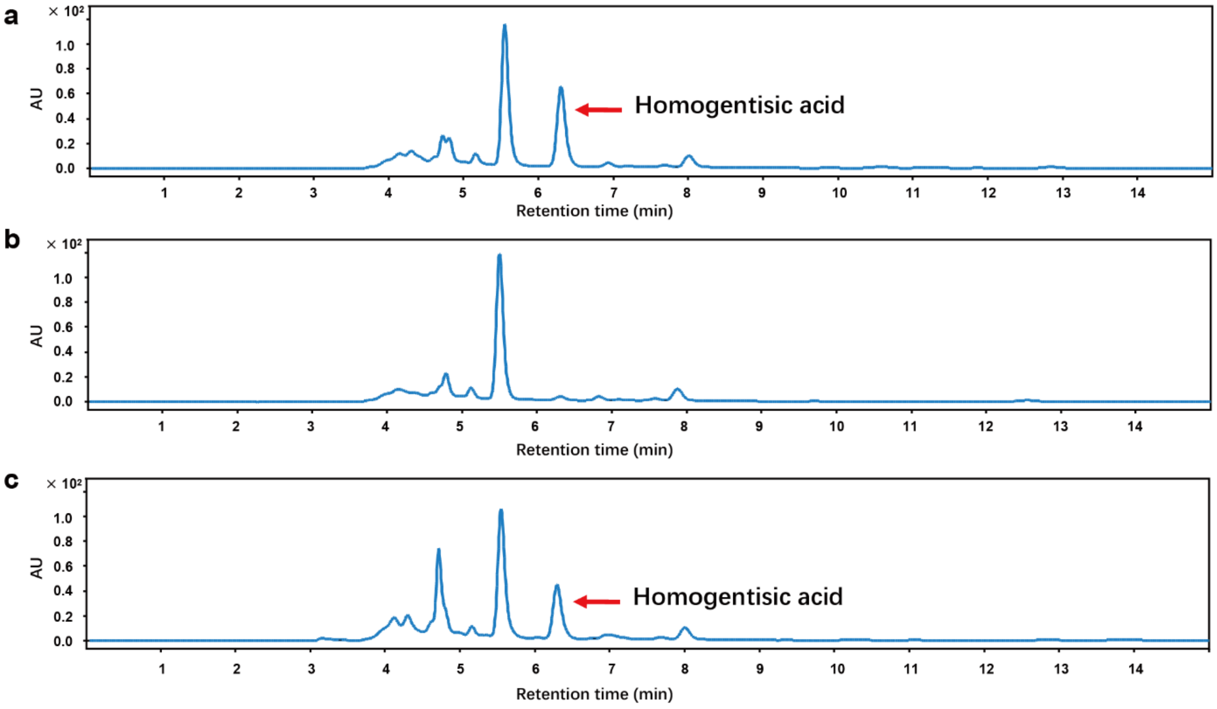

Figure S4. HPLC analysis of supernatants of (a) BMB171 + homogentisic acid, (b) BMB171, and (c) BMB181. Homogentisic acid was indicated by red arrows.

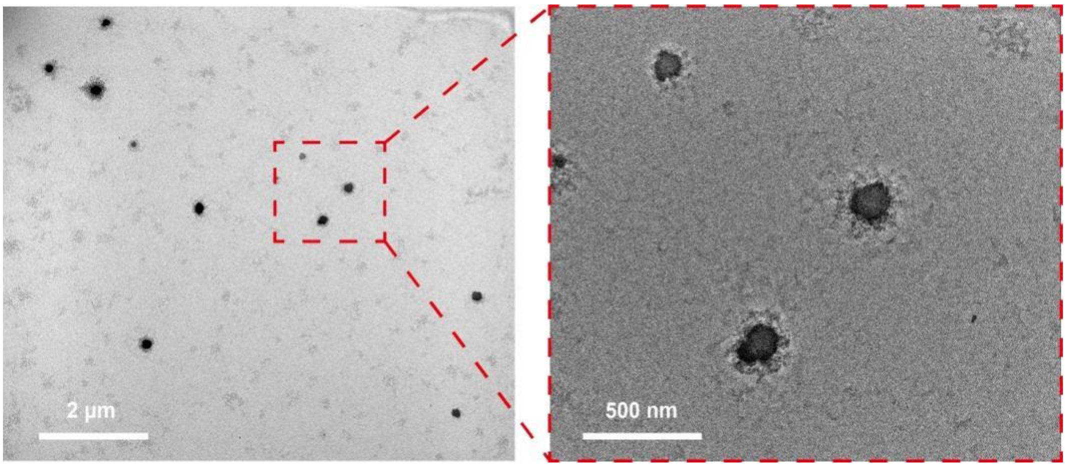

Figure S5. TEM images of melanin produced by BMB181.

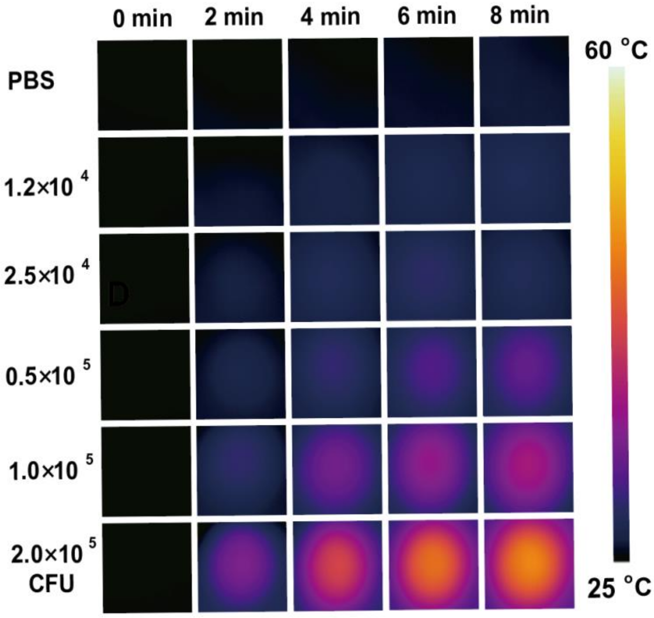

Figure S6. Thermal images of BMB181 dispersions at different concentrations (0, 1.2×10^4, 2.5×10^4, 0.5×10^5, 1.0×10^5, and 2.0×10^5 CFU) upon 808 nm laser irradiation (1.5 W cm^{-2}, 8 min).

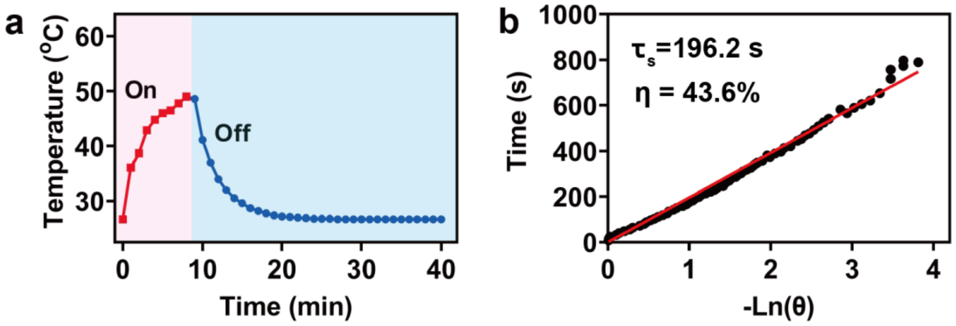

Figure S7. a) Photothermal effects of BMB181 dispersion upon 808 nm laser irradiation (1.5 W cm^{-2}). b) The fitting linear curve of cooling time versus the negative natural logarithm of the driving force temperature during the cooling period of BMB181 dispersion in (a).

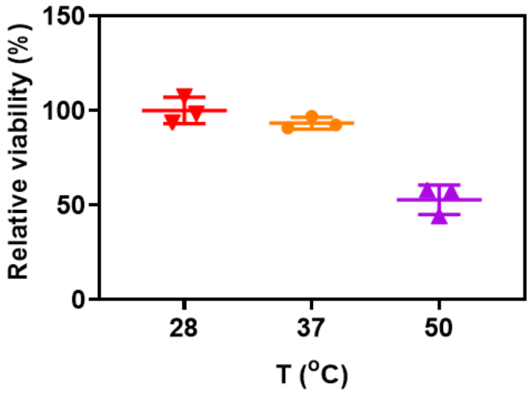

Figure S8. Relative viability of BMB181 after heating at diverse temperatures (28 ºC, 37 ºC, and 50 ºC) for 5 min (n = 3). The data are presented as mean value ± SD.

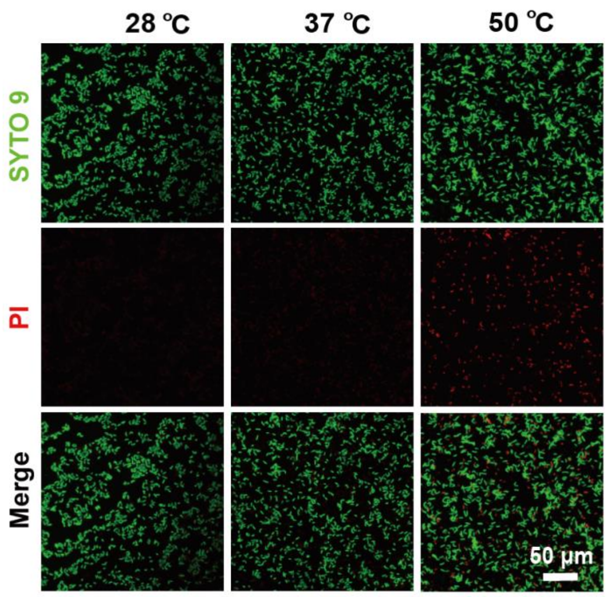

Figure S9. Fluorescence microscopy images of BMB181 co-stained with SYTO9 (green, live bacteria) and PI (red, dead bacteria) dyes after different temperature treatments (28 ºC, 37 ºC, and 50 ºC).

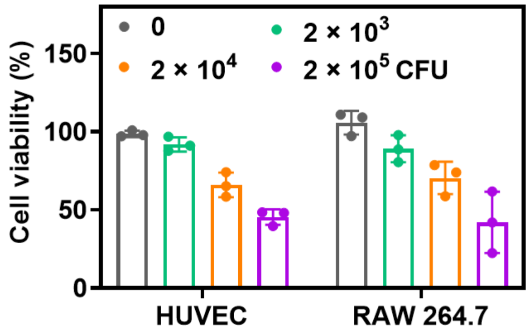

Figure S10. Cell viability of HUVEC and RAW264.7 cells after incubation with BMB181 dispersions at different concentrations (0, 2.0 × 10^3, 2.0 × 10^4, and 2.0 × 10^5 CFU) for 24 h (n = 3). The data are presented as mean value ± SD.

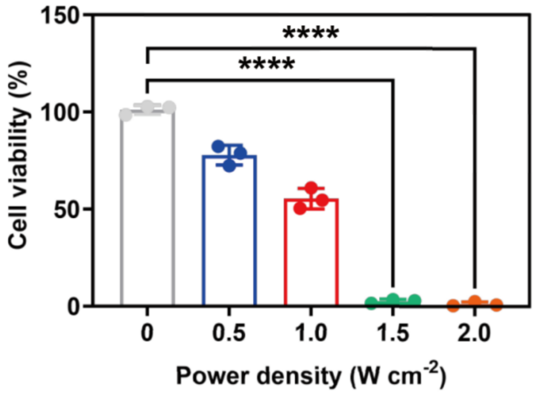

Figure S11. The cell viability of 4T1 cells treatment with BMB181 (2.0 × 10^5 CFU) under 808 nm laser irradiation at different power densities (0, 0.5, 1.0, 1.5, and 2.0 W cm^{-2}) for 8 min (n = 3). The data are presented as mean value ± SD. Statistical significance was calculated using Two-tailed Student's t-test, $^{****}p < 0.0001$.

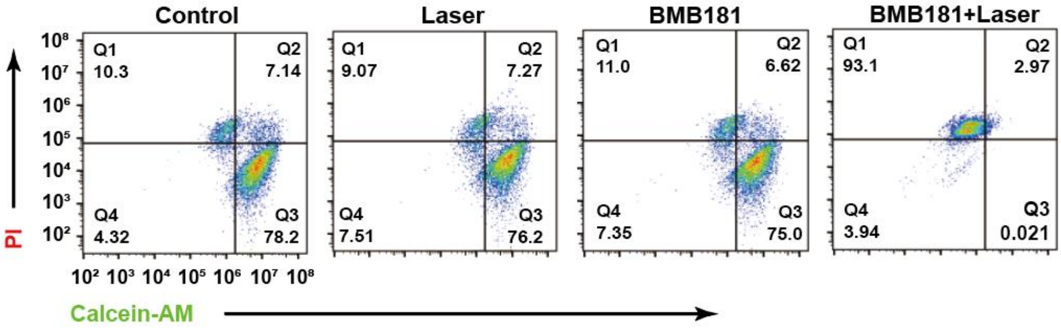

Figure S12. Flow cytometry analysis of 4T1 cells stained with Calcein-AM and PI after treatment with different processes, including Control, Laser, BMB181, and BMB181 + Laser.

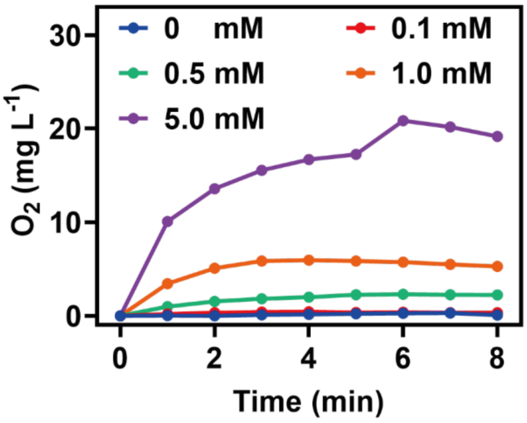

Figure S13. Time-dependent O_2 generation by BMB181 (2.0×10^5 CFU) treatment with H_2O_2 at different concentrations (0, 0.1, 0.5, 1.0, and 5.0 mM).

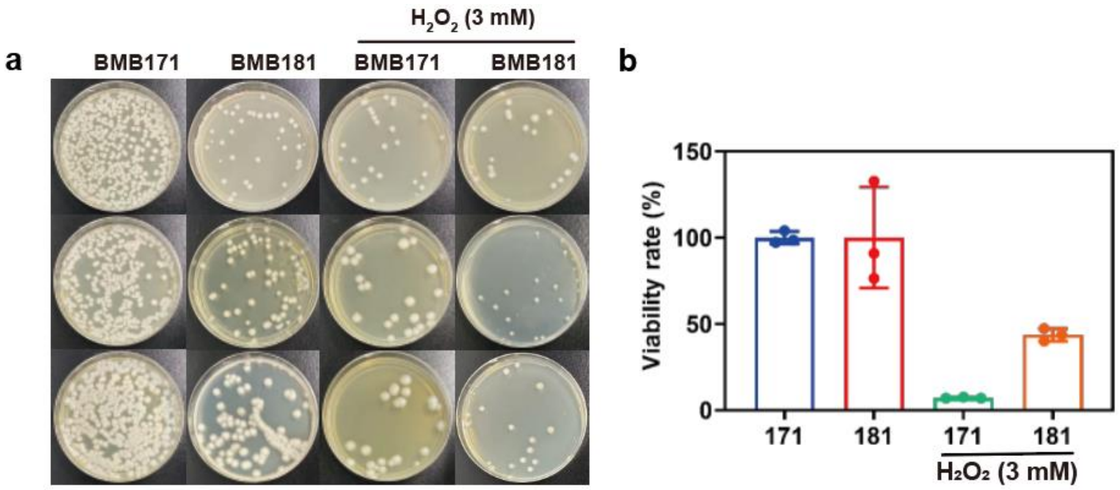

Figure S14. a) Digital photos of bacterial colonies of BMB171 and BMB181 treatment with (right) and without (left) H_2O_2 (3 mM) for 6 h. b) The quantification of bacterial viability of BMB171 and BMB181 strains from (a) (n = 3). The data are presented as mean value ± SD.

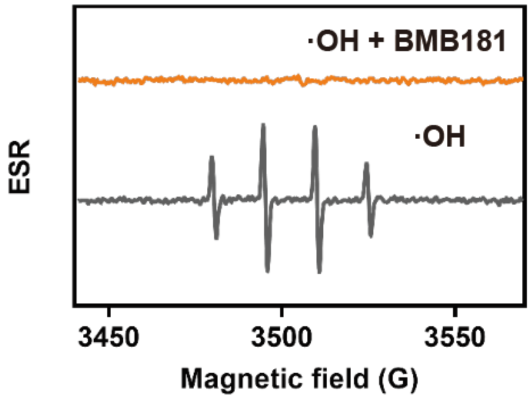

Figure S15. a) ESR spectra displaying •OH elimination of BMB181.

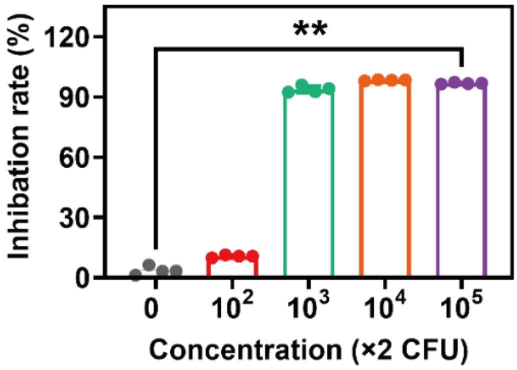

Figure S16. •OH scavenging by BMB181 at different concentrations (0, 2.0 × 10^2, 2.0 × 10^3, 2.0 × 10^4, and 2.0 × 10^5 CFU) (n = 4). The data are presented as mean value ± SD. Statistical significance was calculated using Two-tailed Student's t-test, $^{**}p < 0.01$.

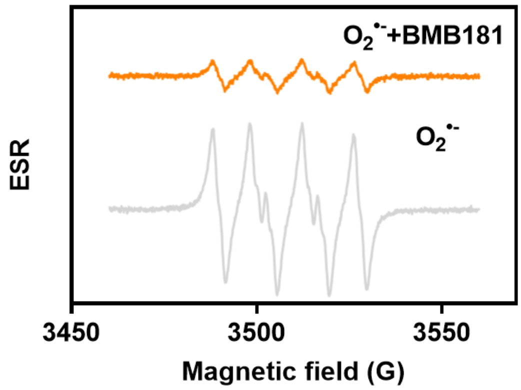

Figure S17. ESR spectra demonstrating $O_2^{\bullet-}$ elimination of BMB181.

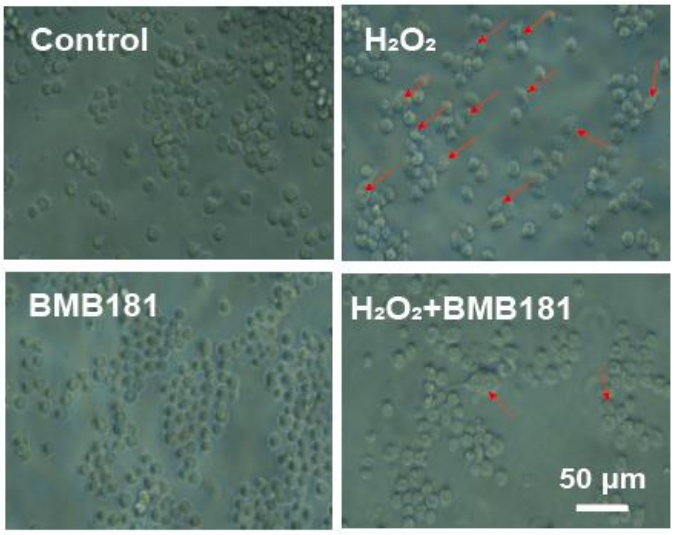

Figure S18. Micrographs of RAW264.7 cells after various treatments. The red arrow indicates the activated macrophages that differentiate into pseudopods.

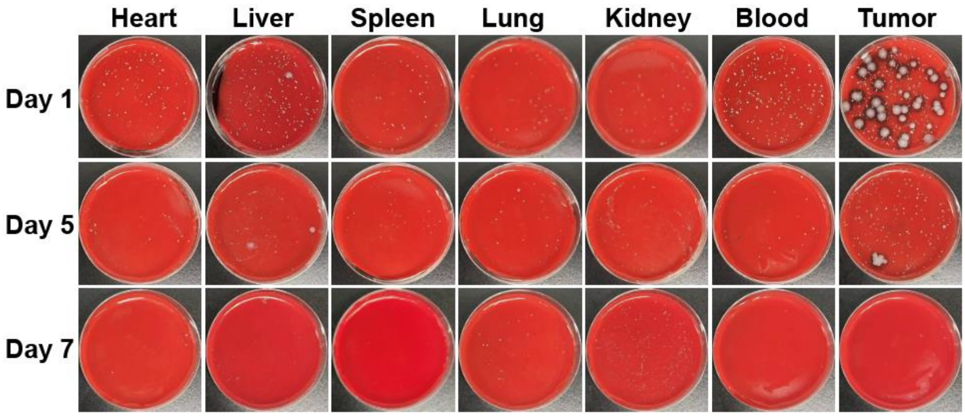

Figure S19. The distribution of BMB181 in the heart, liver, spleen, lung, kidney, blood, and tumor tissue at pointed times.

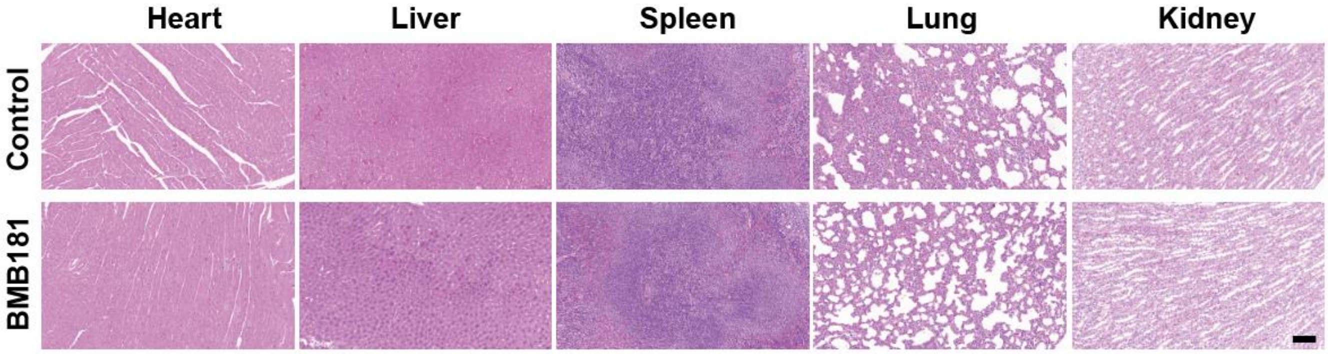

Figure S20. H&E staining of the major organs including heart, liver, spleen, lung, and kidney after different treatments for 7 days. Scale bar: 100 μm.

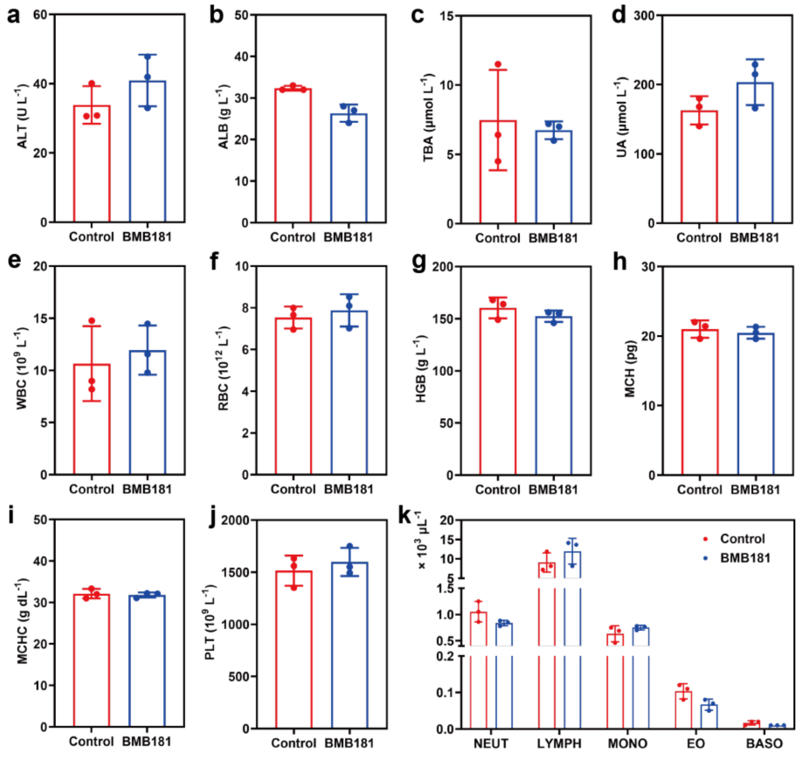

Figure S21. Biosafety study of BMB181 in vivo. (a-d) Blood biochemical analysis, including alanine aminotransferase (ALT), albumin (ALB), total bile acid (TBA), and uric acid (UA) after injection with BMB181 at 7 days (n = 3). (e-k) Routine blood analysis, including white blood cells (WBC), red blood cells (RBC), hemoglobin (HGB), mean corpuscular hemoglobin (MCH), mean corpuscular hemoglobin concentration (MCHC), platelet (PLT), neutrophils (NEUT), lymphocyte (LYMPH), monocyte (MONO), eosinophil (EO), and basophil (BASO), after injection with BMB181 at 7 days (n = 3). The data are presented as mean value ± SD.

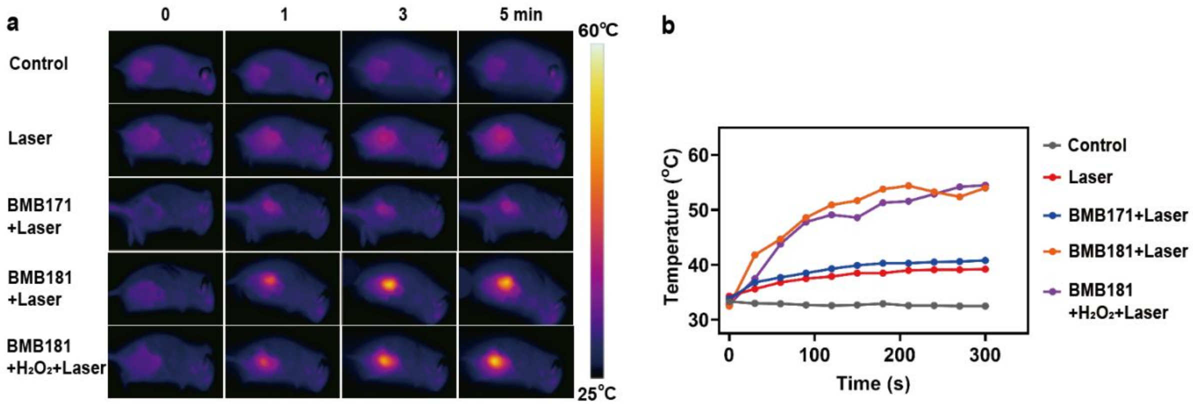

Figure S22. (a) Representative infrared imaging of tumor-bearing mice in different groups, including Control; Laser (1.5 W cm^{-2}, 5 min); BMB171 + Laser (2.0 × 10^5 CFU, 1.5 W cm^{-2}, 5 min), BMB181 + Laser (2.0 × 10^5 CFU, 1.5 W cm^{-2}, 5 min); and BMB181 + H_2O_2 + Laser (2.0 × 10^5 CFU, 1.5 W cm^{-2}, 5 min) and (b) the corresponding temperature change curves.

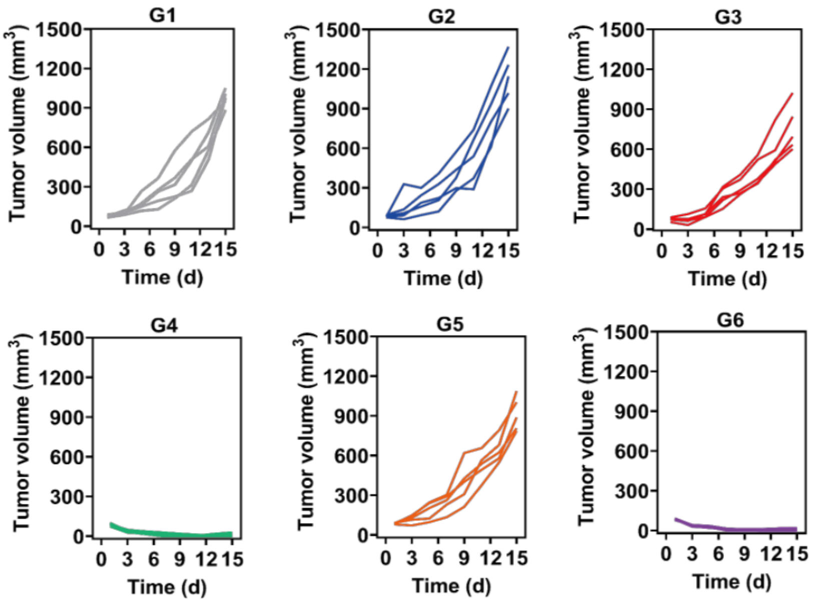

Figure S23. Individual tumor growth curves of 4T1 tumor-bearing mice after different treatments, including G1: Control; G2: Laser; G3: BMB181; G4: BMB181 + Laser; G5: H_2O_2 + Laser; G6: BMB181 + H_2O_2 + Laser (n = 5).

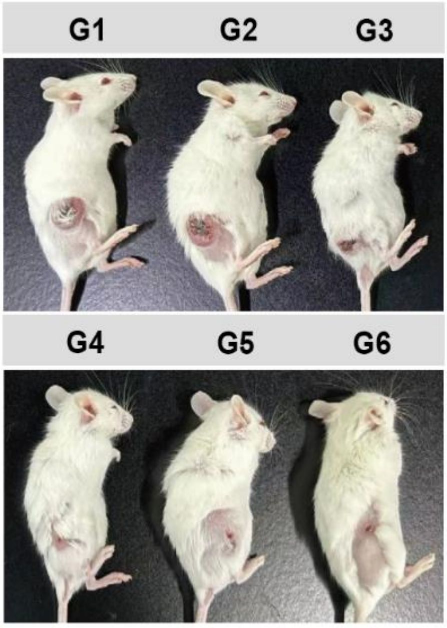

Figure S24. Representative photographs of mice on 14 days in different groups, including G1: Control; G2: Laser; G3: BMB181; G4: BMB181 + Laser; G5: H_2O_2 + Laser; G6: BMB181 + H_2O_2 + Laser (n = 5).

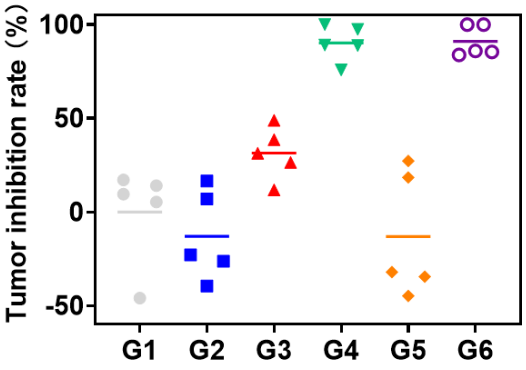

Figure S25. Tumor inhibition rate under different treatments, including G1: Control; G2: Laser; G3: BMB181; G4: BMB181 + Laser; G5: H_2O_2 + Laser; G6: BMB181 + H_2O_2 + Laser (n = 5).

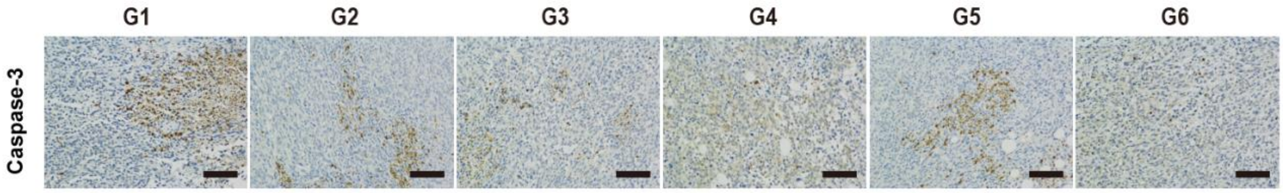

Figure S26. Immunohistochemistry staining of caspase-3 of tumor sections in different groups, including G1: Control; G2: Laser; G3: BMB181; G4: BMB181 + Laser; G5: H_2O_2 + Laser; G6: BMB181 + H_2O_2 + Laser. Scale bar: 100 μm.

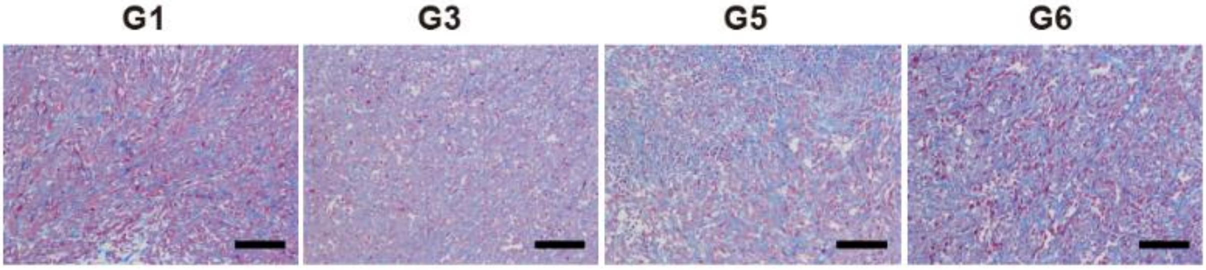

Figure S27. Masson's trichrome staining of tumor sections in different groups, including G1: Control; G3: BMB181; G5: H_2O_2 + Laser; G6: BMB181 + H_2O_2 + Laser. Scale bar: 100 μm.

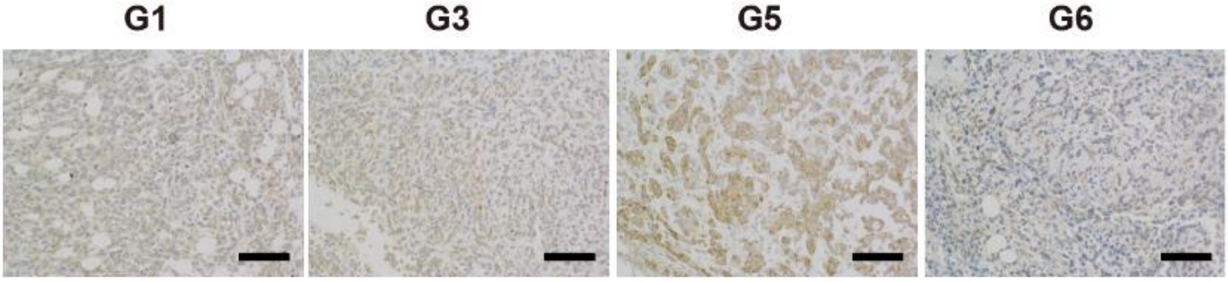

Figure S28. Representative immunohistochemistry staining of TNF-α of tumor sections in different groups, including G1: Control; G3: BMB181; G5: H_2O_2 + Laser; G6: BMB181 + H_2O_2 + Laser. Scale bar: 100 μm.

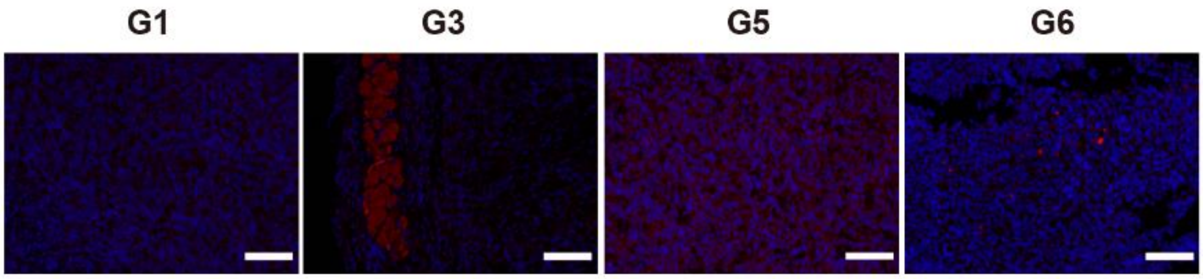

Figure S29. Immunofluorescence staining of IL-6 of tumor sections in different groups, including G1: Control; G3: BMB181; G5: H_2O_2 + Laser; G6: BMB181 + H_2O_2 + Laser. Scale bar: 100 μm.

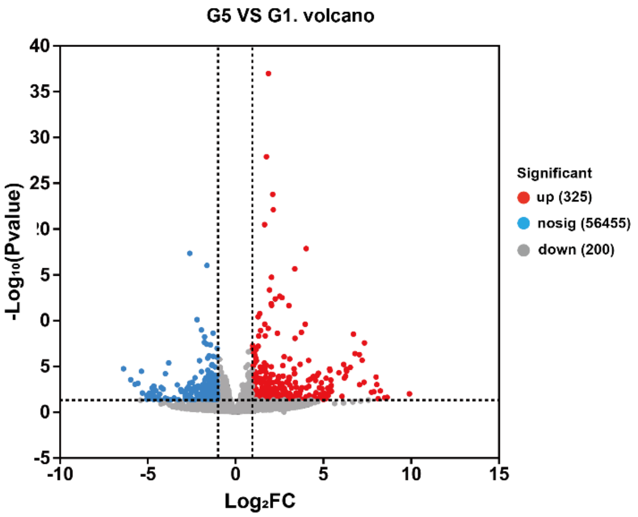

Figure S30. The volcano map showing the identified upregulated and downregulated genes after H_2O_2 + Laser treatment (| Fold Change | ≥ 2, p < 0.05).

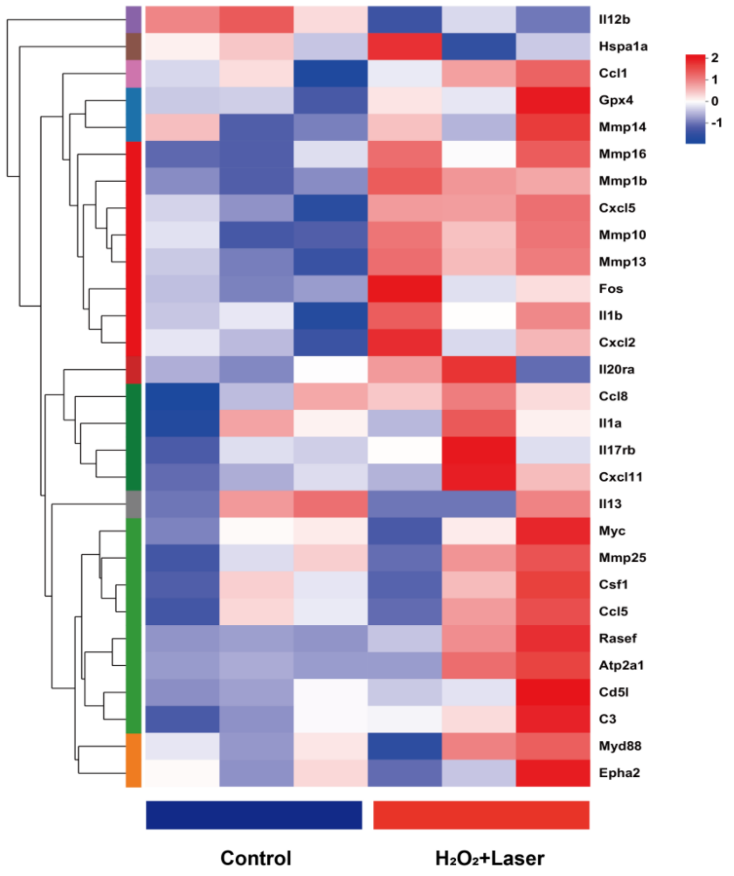

Figure S31. Heat map of the DEGs from Control (G1) and H_2O_2 + Laser (G5) groups (| Fold Change | ≥ 2, p < 0.05).

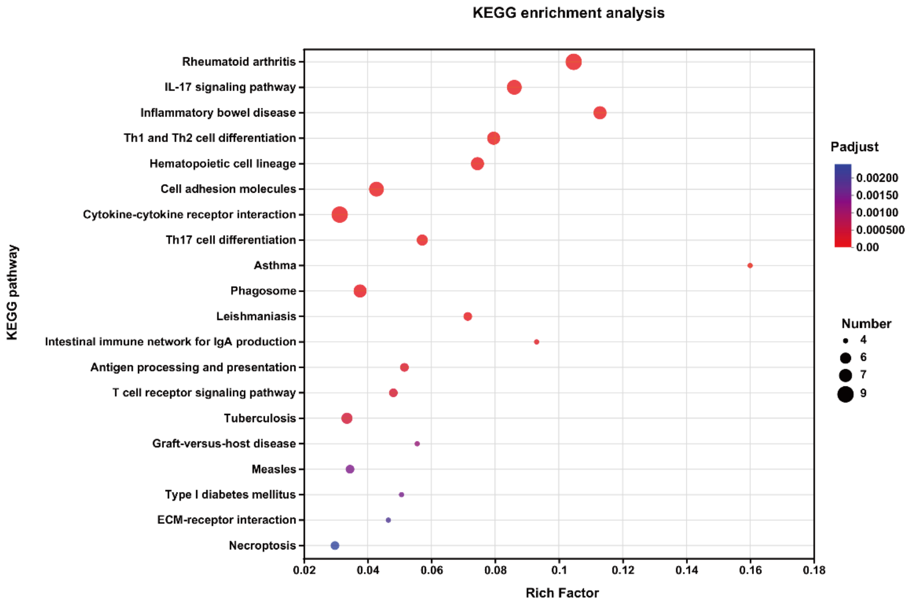

Figure S32. KEGG pathways enrichment analysis of the top 20 pathways of the DEGs from H_2O_2 + Laser (G5) and BMB181 + H_2O_2 + Laser (G6) groups (| Fold Change | ≥ 2, p < 0.05).

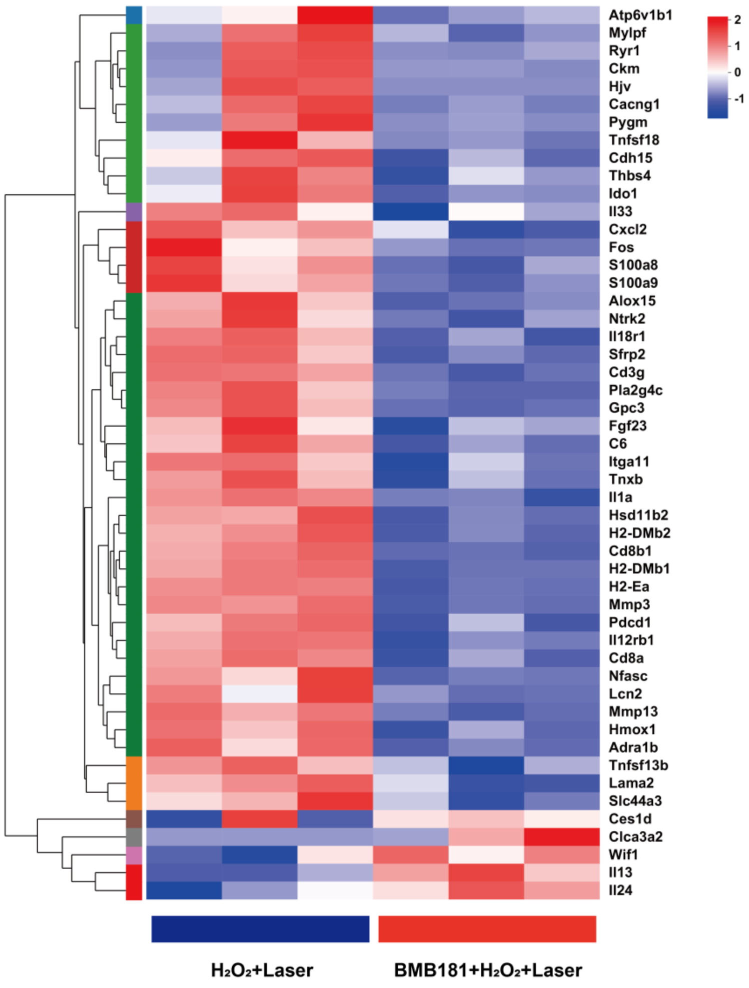

Figure S33. Heat map of the DEGs from H_2O_2 + Laser (G5) and BMB181 + H_2O_2 + Laser (G6) groups (| Fold Change | ≥ 2, *p* < 0.05).

Supplementary Sequence: Sequence alignment of homologous dioxygenase (HmgA) proteins from BMB171 and BMB181 strains.

BMB171 ……GTGKIETMFGTIHYRKGDYVTIPIGTIYRVIPDEGETKFLVVEANS

BMB181 ……GTGKIETMFGTIHYRKGDYVTIPIGTIYRVIPDEGETKFLVVEANS

BMB171 QITTPRRYRNEYGQLLEHSPFCERDLRGPEKLETYDEKGEFVVM

BMB181 QITTPRRYRNEYGQLLEHSPFCERDLRGPEKLETYDEKGEFVVM

BMB171 TKSRGYMHKHVLGHHPLDVVGWDGYLYPWVFNVEDFEPITGR

BMB181 TKSRGYMHKHVLGHHPLDVVGWDGYLYPWVFNVEDFEPITGR

BMB171 IHQPPPVHQTFEGHNFVICSFVPRLYDYHPESIPAPYYHSNVNSDE

BMB181 IHQPPPVHQTFEEHNFVICSFVPRLYDYHPESIPAPYYHSNVNSDE

BMB171 VLYYVEGNFMSRKGVEEGSITLHPSGIPHGPHPGKTEASIGKKET

BMB181 VLYYVEGNFMSRKGVEEGSITLHPSGIPHGPHPGKTEASIGKKET

BMB171 LELAVMIDTFRPLRIVKQAHETEDEKYMYSWIEQGSYTVK......

BMB181 LELAVMIDTFRPLRIVKQAHETEDEKYMYSWIEQGSYTVK......

The genomic DNA sequencing results of homologous dioxygenase (HmgA) encoding BMB171 and BMB181 strains shows that compared with BMB171 strain, the glycine at the residue of HmgA 272 in BMB181 strain is replaced by glutamate.[1] The BMB181 mutation site is highlighted.

Reference

[1] W. J. Yang, L. F. Ruan, J. M. Tao, D. H. Peng, J. S. Zheng, M. Sun, Front. Microbiol. 2018, 9, 2242.

Author Contributions

X.G., W.F., and Y.C. conceived the study. Y.Z., X.D., and W.Y. synthesized and characterized the bacteria. M.C., B.G., H. C, W.W., and. J.J. conducted in vitro and in vivo experiments. M.C., W.W., and C.W. analyzed the data. M.C. wrote the original draft. W.F., D.Y., and Y.C. revised the paper. All authors discussed the experimental procedures and results.
